# Challenges in mass drug administration for treating lymphatic filariasis in Papua, Indonesia

**DOI:** 10.1186/1756-3305-3-70

**Published:** 2010-08-11

**Authors:** Navneet Bhullar, Jacob Maikere

**Affiliations:** 1 Médecins Sans Frontières - Operational Center Brussels (OCB), Rue Dupre 94, Brussels 1090, Belgium

## Abstract

**Background:**

The World Health Organization (WHO) Global Program to Eliminate Lymphatic Filariasis relies on mass drug administration (MDA) of two drugs annually for 4 to 6 years. The goal is to reduce the reservoir of microfilariae in the blood to a level insufficient to maintain transmission by the mosquito vector. In 2008, the international medical aid organization Médecins Sans Frontières (MSF) performed the first round of a MDA in the high-burden area of Asmat district, in Papua, Indonesia. We report the challenges faced in this MDA on a remote Indonesian island and propose solutions to overcome these hurdles in similar future contexts.

**Results:**

During the MDA, we encountered difficult challenges in accessing as well as persuading the patient population to take the antifilarial drugs. Health promotion activities supporting treatment need to be adapted and repetitive, with adequate time and resources allocated for accessing and communicating with local, seminomadic populations. Distribution of bednets resulted in an increase in MDA coverage, but it was still below the 80-85% target.

**Conclusions:**

MDA for lymphatic filariasis is how the WHO has planned to eliminate the disease from endemic areas. Our programmatic experience will hopefully help inform future campaign planning in difficult-to-access, high-burden areas of the world to achieve target MDA coverage for elimination of lymphatic filariasis.

## Background

Lymphatic filariasis (LF) is a parasitic disease transmitted by mosquito bites which causes disability and adversely impacts the economy of the developing countries where it is endemic. LF is the fourth most common cause of disability worldwide [1]. It is caused by *Wuchereria bancrofti, Brugia malayi*, or *Brugia timori*. These parasites reside in lymphatic channels or lymph nodes where they remain viable for more than two decades. *W. bancrofti *is the most widely distributed, affecting an estimated 115 million people throughout the tropics and subtropics. The World Health Organization (WHO) estimates that 120 million people are currently infected and more than 1 billion people are at risk in 83 countries [1]. Approximately 40 million people are seriously incapacitated and disfigured by the disease [1,2].

LF disease transmission can be stopped through a feasible, effective, and relatively inexpensive prevention strategy through mass drug administration (MDA) of two oral drugs to at-risk populations once a year [3-5]. These drugs kill the microfilariae in an infected patient's blood so that mosquitoes cannot transmit the disease to others. This MDA-based strategy of transmission interruption is part of the WHO's Global Program to Eliminate Lymphatic Filariasis [6].

Filariasis is an eradicable disease of high prevalence among several of the islands of Indonesia. Due to the high prevalence and remote distribution and diversity of filarial infections, filariasis elimination in Indonesia is a major challenge [7].

In 2008, the international medical aid organization Médecins Sans Frontières (MSF) offered to carry out the first round of a MDA in the district of Asmat, Papua, Indonesia, where LF was found to be endemic. We report here our experience and the challenges faced in this MDA against LF in Asmat over an 11-week period from July to September 2008.

## Results

### Outcomes of the MDA Campaign

The first MSF filariasis campaign took place in Asmat District from July 9, 2008, the date of the first MDA activity, to September 22, 2008, date of the last MDA activity. All the 7 subdistricts were covered, comprising 147 villages visited over 28,000 km^2^.

A total of 36,478 people took the drug treatment, giving an average coverage of 58% of the targeted population. The teams spent a total of about 1,000 hours on boats, consuming a total of 24 tons of fuel, representing an average of 1.5 people treated per liter of fuel used in this campaign.

### Program Challenges

#### Accessing and communicating with populations and workers

Because of their remoteness, the villages were not often visited by outsiders and were accessible only by boat. No radio, television, or newspaper media exist, ruling out advertising educational campaigns through the mass media. Due to this difficulty of accessing local populations, villagers were visited by local health workers about once a month. Workers went to the villages a few weeks before the MDA campaign, and the upcoming campaign was mentioned. Attempts were sometimes made to send out dates of the MDA through the District Health Office (DHO), but these notices were often not distributed.

Local health workers however did not perform much health promotion, with most of this task handled by MSF. For example, some puskesmas (local health center) staff did not receive invitations for training since postal service is nonexistent in the villages of Asmat, though radio communication to the puskesmas was present at times. Messages were often conveyed word-of-mouth and depended on the next boat that arrived from Agat, the capital.

In the village of Yahoi in Fayit, there had been some deaths from snakebites the previous 2 years, and the villages asked for antivenom. Later, on the day of MDA, some villagers were agitated that MSF was treating filariasis in people who had no obvious signs of the disease, when they needed basic health care for diseases like malaria and diarrhea. The physician happened to be there to explain to the people that filariasis can be eradicated with these drugs, but means to eradicate malaria and diarrhea, for instance, have not been found yet.

MSF faced program difficulties in Sawa Erma, the subdistrict with the largest population. Rumors in the subdistrict included, "you drown if you take the drugs" and "someone died in Agat after taking the drugs." With the vastness of this subdistrict (Sawa Erma) and a little under 2 weeks allotted to it, we experienced a marked fall in drug administration compliance, averaging only 37% population coverage. Convincing the local population was difficult, as they had little trust in the MDA program. To try to remedy this, the village chief in Sawa took the drugs to demonstrate their safety (a practice often repeated throughout the campaign), but the people watching still would not register to take the drugs. Talking to reassure them was also not helpful. This difficulty was not encountered in other subdistricts.

In other subdistricts, for instance in Atsy and Pantai Kasuari, where there was insufficient health care, people came to us and requested health care. Some people narrated the number of infant deaths from diarrhea in the previous year, or the high number of asthma cases in a village. When we explained that we were just working on filariasis in collaboration with the DHO, it was hard to gain trust. The population, rightly, had concerns about other diseases that were life-threatening. In the face of health care difficulties such as these, just telling the needy villagers we would pass the word along, and that our task here was only MDA, may have sounded superficial. This has been the experience in other MDA campaigns as well [8].

The village headmen were often absent, so finding a person the villagers trusted, who would help convince them, was also a challenge. Villagers were sometimes absent from villages, lowering coverage. The reasons given were "they have gone to Agat" or "they are in the forest looking for food." Visits to bewaks (temporary river shelters) were not always fruitful since people were in the forest.

Population numbers were often unreliable. The numbers provided by the DHO were projections from the 2000 census when Asmat was still part of the district of Merauke. No newer numbers were available at the time of the intervention. These numbers were found to be inflated when one talked to village authorities and tallied numbers. For instance, in the village of Yomoth, our population figure was 647, the village head told us 502, and a village elder told us only about 300 people actually lived in the village. A total of 180 people were treated with the drugs. Drug coverage could not therefore be accurately determined.

One or two hours of health promotion, depending on village size, was not always sufficient to convince people of the importance of taking the drugs. Establishing trust took more time and effort than anticipated.

#### Mapping of bewaks

No maps were available indicating each village's bewaks, probably because they are temporary structures. But accessing people living in bewaks was crucial to the success of a campaign of this nature, which necessitates high coverage. Knowing the locations of bewaks was difficult, especially in the largest subdistrict of Sawa Erma. Information on bewaks was not available, nor attempted to be obtained, at the time of MDA planning. Exploration was not done in this subdistrict since mobile clinics had been conducted here, but the populations who came for mobile clinics had sought medical attention for other ailments.

The bewaks tended to be scattered and only villagers could take us to them, something not factored into the time frame of the campaign. In the time table for the campaign, one day was allotted for each village of a subdistrict. To return to base by 5:00 PM, a team may sometimes have to leave 2 hours beforehand, and that left no time to visit a large portion of the populations in bewaks. Also, bewaks were often inaccessible even when there was time to visit them for drug distribution.

#### Treatment incentives

Population turnout and compliance was best in the 4 subdistricts where bednets were distributed (Table 1). People often rowed long distances from bewaks and even came to our base village to take drugs when these people had been absent at the time of drug distribution in villages.

**Table 1:** Population coverage of filariasis MDA in 7 subdistricts of Asmat district, Papua, Indonesia, 2008

Subdistrict	Total population*	Target population^†^	MDA coverage,*n (%)	Bednets used
Agats^‡^	8349	6679	5207 (78.0)	0

Akat	6465	5172	2964 (57.3)	0

Sawa Erma	15,204	12,169	4263 (35.0)	0

Suator	8139	6511	3958 (60.8)	1404

Atsy	14,867	11,894	7730 (65.0)	2444

Fayit	6939	5551	4085 (73.6)	1295

Pantai Kasuari	16,147	12,918	7751 (60.0)	2400

TOTAL	76,110	60,893	35,958 (59.1)	7543

MDA, mass drug administration

*Only villages originally planned for MDA coverage and reached were included in this analysis.

**^†^**Target population calculated as 80% of total population.

^‡^In Agats subdistrict, the village of Bis Agats received multiple sweeping rounds (since it was near MSF headquarters and thus most accessible) and numerous visitors from neighboring villages, resulting in very high coverage (157%) that could possibly skew the overall coverage picture of Agats. If Bis Agats is excluded, then total population = 5758, target population = 4606, coverage n = 1952, and % coverage = 42.4%.

Staying overnight in villages after drug distribution so adverse events could be examined and treated helped increase compliance. The adverse events noted were fever, nausea, and vomiting. When the team could stay on a little later after the end of drug distribution (being close to or in the base village), people's questions were answered, and adverse events could be ascertained and treated appropriately.

## Discussion

### Lessons Learned

Lessons learned to potentially apply to future MDA campaigns include:

1. In remote locations with no media presence and with the difficulty of transportation to remote villages and their bewaks, accurate mapping must be carried out first.

2. Adequate time must be allocated for studying the populations and devising effective health promotion techniques in collaboration with local health authorities and respected figures in the area. Such feedback is crucial. Visiting villages at least once before the start of the campaign to introduce it, meeting with village elders and establishing rapport, and returning to repeat and announce the campaign date may be effective strategies.

3. Adequate time must be allotted to the actual MDA campaign so that (a) geographically most if not all the population can be reached; (b) villages can be revisited, if possible, to allow time for people to come from places such as bewaks; (c) lessons learned from villages covered early on in the campaign can be applied meaningfully to villages visited later; and (d) unforeseen events such as inappropriate weather or fuel shortages can be dealt with easily.

4. Incentives to the LF-affected populations may be useful in improving compliance. These can be materials (eg, bednets) or human resources (eg, holding free clinics to treat other ailments if time permits).

5. Polyvalent teams are more efficient (for both health promotion and MDA activities):

• The same team should do health promotion and MDA activities in one village

• Optimal team is composed of one nurse, one health promoter, and one logistician

• About 1.5 hours needed for health promotion activity in one village.

• A MDA team (3 people) can cover approximately 60 patients/hour (suitable for a village), up to 500 people/day

6. Local people within the staff can help reassure the populations during MDA

### Strategies for Accessing Hard-to-Reach Populations

Reaching the seminomadic populations in Asmat for LF MDA was a challenge, as was convincing people to take the drugs. Coupled with remoteness of location, and no readily utilizable mass media, an effective MDA requires sufficient time, repeat visits, and high emphasis on health promotion and establishing trust with local populations. Eliciting direct help and guidance from local, respected, high-standing villagers and sometimes missionaries and local pastors can be useful. Taking the necessary time and effort to establish understanding and trust should be borne out in future MDA campaigns. Coupled incentives to populations, such as distributing bednets, may help increase compliance.

## Conclusions

Preventative MDA for a disease such as filariasis, which is chronic and has seemingly little effect on day-to-day life, is a major challenge. Filariasis affects parts of the world that are not industrialized and where people may not be generally aware of disease risk. Since MDA is done among the general population, the majority of whom have no symptoms of filariasis, compliance is the major hurdle. In this experience, when bednets were given with the drugs, compliance increased, indicating the potential importance of such incentives to populations when a non-life-threatening disease such as filariasis is targeted for intervention.

## Methods

### Intervention Setting and Characteristics

Papua is the second largest island in the world and is governed by two countries: Papua New Guinea in the east and Indonesia in the west. The district of Asmat in western Papua has an area of about 28,000 km^2 ^and population of over 75,000. Asmat district has 7 subdistricts: Agats, Akat, Sawa Erma, Suator, Atsy, Fayit, and Pantai Kasuari.

**Figure 1:**
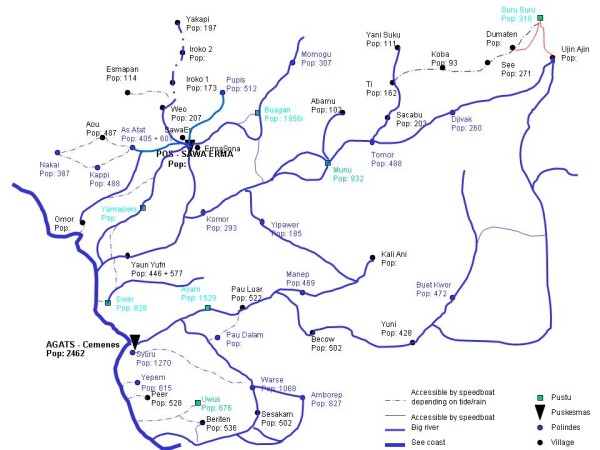
**Map of Asmat district**.

MSF provided mobile clinics in Agats, Akat, and Sawa Erma from 2006 to 2008. MSF staff were based in Agat, the center of Agats and the capital of Asmat, and in the village of Pos in Sawa Erma (Figure 1). The MDA was carried out in the following order: Agats to Akat to Sawa Erma, and then Atsy, Suator, Fayit, and Pantai Kasuari.

Off the Arafura Sea, Asmat district is remote, with access to villages only by boat via rivers that jut inland. The area is mainly swamp land and lacks electricity, roads, agriculture, and other modern facilities. The population is relatively poor and primarily has a middle-school level of education, with men tending to be educated at a higher level than the women. Fishing and foraging for wood for trade are the main occupations.

**Figure 2:**
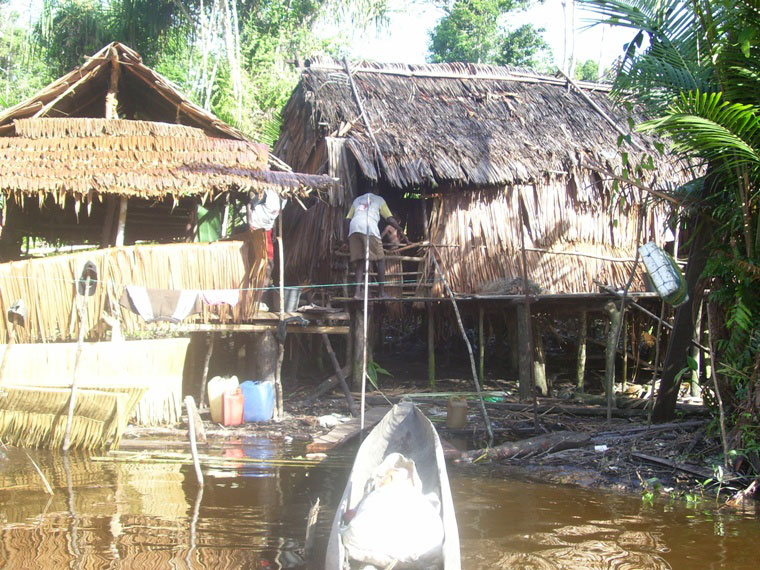
**Bewak**. Credit: Navneet Bhullar/MSF.

**Figure 3:**
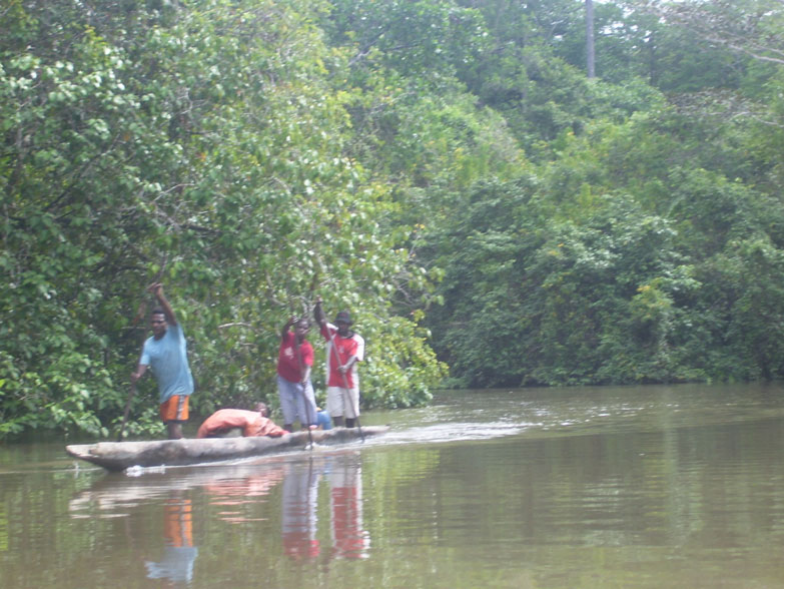
**Kolekole**. Credit: Navneet Bhullar/MSF.

The village population is divided between the actual village, and bewaks, temporary shelters built on rivers where groups of villagers go for a few months each year to forage in the forest for food and wood (Figure 2). Bewaks belong to particular villages. Families travel in kolekole, which are dug-out, narrow, long wooden canoes typically rowed by two people (Figure 3).

Local health care is offered in the form of health centers called puskesmas, with one each in a subdistrict capital, and village health posts called pustus, run by a nurse and/or midwife. The DHO (also known as Dinas Kesehatan) is based in Agat, the district capital.

Beginning in November 2006 over 2 years, MSF helped provide basic health care through mobile clinics. In February 2008, MSF launched a study comparing the performance of three malaria rapid diagnostic tests with that of microscopy (gold standard). A total of 492 patients were included in this study from 3 subdistricts (Sawa Erma, Agats, and Akat) in which 245 (49.8%) were children under 5 years of age. Most of the patients came from Sawa Erma (60.2%). Microfilariae were found among 10 patients: 6 positive for *W. bancrofti*, 2 positive of *B. malayi*, and 2 mixed between *W. bancrofti *and *B. malayi*. All were from Sawa Erma subdistrict in 5 villages (Bu Agani, Aou, Kappi, Weo, and Mumugu), which confirmed the clinical findings. The microfilaria rate was thus 2.03%, which exceeds the criteria of the Ministry of Health (MOH) and WHO (≥1%) to declare an area as endemic [9].

In Asmat, both limb elephantiasis and hydrocele (enlargement of the scrotum with fluid due to obstruction of lymphatic drainage by the worm) are symptomatic manifestations of chronic filariasis. Occasionally, unilateral breast enlargement has also been observed.

Because the district did not have the required budget to carry out a MDA campaign for filariasis, in 2008 MSF offered to perform the first round of MDA. The MOH and DHO committed to continuing the campaign for the remaining four rounds over the following 4 years.

### Medical and Health Promotion Teams and Strategy

**Figure 4:**
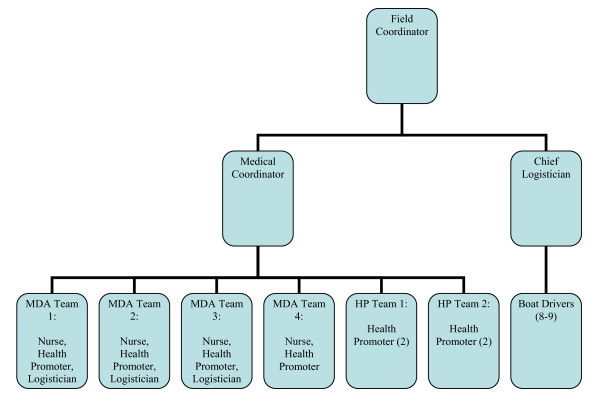
**Filariasis MDA team organizational chart, Asmat district, Papua, Indonesia, 2008**. MDA, mass drug administration; HP, health promotion.

The filariasis MDA team was comprised of one field coordinator, one physician (medical coordinator), 4 nurses, and 8 health promoters, in addition to the logistics staff (Figure 4). The medical teams were comprised of one nurse, one health promoter, a logistician, and a boat driver. The health promotion teams each had two health promoters and a boat driver. The complete MDA team of initially 27 people was expanded to 29 to cover the last 2 subdistricts.

The health promotion team traveled to villages and spoke with the village chief or another respected person. They then gathered the villagers at a common place and introduced themselves, and described filariasis and the importance of eradicating it. The team explained that all villagers (except pregnant and lactating women, persons with ongoing fever, and the chronically ill) would be given three drugs for this purpose, two to treat the active and nascent disease (albendazole and diethylcarbamazine) and the third (paracetamol) to reduce side effects. The team used flip charts and leaflets as educational aids, and hung posters explaining the disease cycle and drugs in simple terms. In subdistricts where MSF had not worked before, MSF newsletters were given to the village chief.

Written materials were in Bahasa Indonesia, the national language of Indonesia. The team used microphones to walk around the village announcing the day of the MDA campaign, along with the venue advised by the village elders. The team met with midwives and/or kaders (community health workers) to help with registrations.

One to three days after the health promotion team visited a village (except in the largest subdistrict of Sawa Erma, where longer time sometimes elapsed between health promotion and MDA activities), the MDA team arrived in the morning, set up base at the decided venue, briefly described the disease and treatment again, and proceeded to start MDA. When turnout was low, the team sometimes went door to door. Also, in such cases, they went to the bewaks close by, if accessible. The team was required to leave at a time that would allow them to return to the team base by 5:00 PM (since the boats did not have adequate equipment for navigation in the dark). The base was the central village where all the teams stayed while MDA was done in one subdistrict.

At the end of each subdistrict, sweeping (the process of returning to a population with drugs where adequate coverage was not accomplished the first time) was done in 4 villages with the least coverage on the original MDA day. As an ancillary to the MDA, registration of hydrocele in men and malnutrition in children 2-5 years old was performed. At the end of the campaign, hydrocele repair was to be planned. This was done a few months after the end of the MDA campaign.

In the subdistricts of Atsy, Suator, Fayit, and Pantai Kasuari, one bednet was given to each family administered drugs. Villagers were informed of this distribution on the day of health promotion, and it was made clear that bednets were not a cure for filariasis, but helped prevent malaria and could help with filariasis prevention to some degree as well. The bednets had already been distributed for malaria prevention in the other subdistricts over the last 2 years since MSF had had a presence there.

### Health Worker Training

Conducted by MSF, this first round of the MDA campaign was a collaboration with the DHO. Two doctors from the MOH arrived from Jakarta to help in the training of local health and MSF staff. Doctors from the 7 puskesmas of Asmat were invited, as were some ancillary health staff.

The training lasted one day and covered:

• History of MSF in the area and our past activities

• How MSF decided to carry out the first round of MDA

• MOH policy for implementing the filariasis program

• Pathophysiology, symptoms, diagnosis, complications, prevention, and treatment of filariasis in the international context

• Filariasis in Asmat

• Health promotion strategies

• MDA organization at health center level

• Demonstration of drug distribution and MDA technique

• Adverse reactions of drugs (minimal and minor)

• Methods of reporting drug administration coverage

• Security guidelines

Health promotion training involved educating the patient populations of the ancillary effects of the filariasis drugs, such as the elimination of intestinal helminths and decreases in infant and maternal morbidity. Training of MSF staff was reinforced after 5 subdistricts were completed. Training was also improvised as we encountered patients who complained of limb pain, fever, and itching after drug treatment. The patients were told, for instance, that if adverse events were encountered, it meant the drugs were working (so they would not assume the drugs were making them sick and pass that on to other villagers), and to seek treatment for adverse events in the local health centers.

## Competing interests

The authors declare that they have no competing interests.

## Authors' contributions

NB and JM conceived and designed the study, acquired and analyzed the data, drafted and critically revised the manuscript, and read and approved the final manuscript.
